# Fast Li₂O₂ Electrochemistry Enabled by Co‐N_x_/Co (111) with Optimized Intermediate Adsorption

**DOI:** 10.1002/advs.202510256

**Published:** 2025-07-25

**Authors:** Lili Liu, Chen Wang, Luxin Zhao, Yayun Xiao, Weiwei Fang, Lanling Zhao, Faxing Wang, Yuping Wu

**Affiliations:** ^1^ School of Energy Science and Engineering Nanjing Tech University Nanjing Jiangsu 211816 China; ^2^ Jiangsu Co‐Innovation Center of Efficient Processing and Utilization of Forest Resources College of Chemical Engineering Nanjing Forestry University (NFU) Nanjing 210037 China; ^3^ School of Physics Shandong University Jinan 250061 China; ^4^ Confucius Energy Storage Lab School of Energy and Environment & Z Energy Storage Center Southeast University Nanjing 211189 China

**Keywords:** Co‐N_x_/Co(111), cycling stability, intermediated adsorption, lithium peroxide, lithium‐oxygen batteries

## Abstract

The practical development of Li‐O_2_ batteries (LOBs) urgently needs to explore robust cathode catalysts to boost the sluggish Li_2_O_2_ reaction kinetics and parasitic reactions despite their theoretically high specific energy. Profound understanding of the cathode properties and the battery performance is rather critical in developing rational‐designed electrocatalysts. In this study, a Co‐N_x_/Co (111) decorated N‐doped hierarchical carbon framework (Co‐N_x_/Co@NHCF) is proposed as an efficient cathode in LOBs. Spectroscopic analysis coupled with experimental results suggests that the Co‐N_x_/Co (111) catalytic center can significantly reduce the battery overpotential, and meanwhile, the hierarchical carbon framework ensures rapid mass transportation and provides sufficient space to accommodate Li_2_O_2_ deposition. Density functional theory calculations reveal that the incorporated Co (111) facet can effectively regulate the electronic distribution of N‐carbon, optimize the adsorption of desirable intermediates, and eventually facilitate oxygen reduction reaction/oxygen evolution reaction kinetics. As expected, the Co‐N_x_/Co@NHCF catalyzed LOBs deliver a high discharge/charge capacity of 6.15/ 6.22 mAh cm^−2^ with a columbic efficiency of 98.9%, along with a high rate cycling of 700 h at 0.3 mA cm^−2^. This work provides valuable instruction for the rational design of efficient catalysts for high‐performance LOBs via optimization of the crystal structure and the adsorption of intermediates.

## Graphical abstract

A Co‐N_x_/Co (111) catalytic center demonstrates superior potential in optimizing the adsorption of desirable intermediates via regulating the electronic distribution in the cathode, thus facilitating fast redox kinetics and high rate capability, and stabilized cycling performance for aprotic Li‐O_2_ batteries.

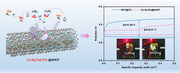

## 1 Introduction

The global new energy market is undergoing unprecedented expansion; however, the performance of commercially available lithium‐ion batteries is nearing their theoretical capacity limits, making them increasingly insufficient to meet the escalating energy demands of next‐generation portable electronics and electric vehicles. Lithium‐oxygen batteries (LOBs) have garnered significant attention as a viable alternative for future high‐energy‐density storage systems, owing to their ultrahigh theoretical energy density (≈3460 Wh kg⁻¹).^[^ ^1^, ^2^ ^]^ The electrochemical processes in LOBs hinge upon the reversible formation and decomposition of lithium peroxide (Li₂O₂) at the cathode (2Li⁺ + O₂ + 2e⁻ ⇌ Li₂O₂). However, the inherently insulating and nearly insoluble nature of Li₂O₂ engenders sluggish oxygen reduction/evolution reaction (ORR/OER) kinetics and progressive cathode passivation during cycling. These intrinsic limitations give rise to substantial challenges, including excessive overpotentials, poor electrochemical reversibility, and severely limited cycle life, thereby hindering their practical implementation.^[^ ^3^ ^–^ ^7^ ^]^

The cathode architecture and catalytic properties play a decisive role in dictating the formation and decomposition pathways of Li₂O₂, thereby critically influencing the overall battery performance. Although toroidal Li₂O₂ via solution pathway is often associated with enhanced discharge capacity, its formation concomitantly induces elevated charge overpotentials, which exacerbate carbon cathode degradation, promote electrolyte decomposition, and facilitate the accumulation of deleterious by‐products such as Li₂CO₃.^[^ ^8^, ^9^ ^]^ Conversely, film‐like Li₂O₂ mediated by the surface formation route enables lower charge overpotentials; however, its progressive surface passivation during discharge obstructs oxygen diffusion channels, leading to premature termination of the discharge process and consequently diminished capacity compared to toroidal Li₂O₂.^[^ ^10^, ^11^ ^]^ Given these intrinsic trade‐offs, the rational design of advanced cathode materials featuring optimized affinity to the discharged intermediates is of great significance in tailing favorable Li_2_O_2_ formation/decomposition pathways and finally realizing high‐performance lithium‐oxygen batteries.

It has been demonstrated that modulating the electronic structure of the catalyst makes huge sense in optimizing the energy barrier of the intermediate generation and accelerating the ORR/OER kinetics.^[^ ^12^, ^13^, ^14^, ^15^, ^16^, ^17^, ^18^ ^]^ For example, a delocalized electronic engineering strategy was employed to fabricate a nitrogen‐doped Ni_5_P_4_ cathode. The nitrogen was proved to remarkably polarize the localized structure, and the energy barrier was lowered for the generation of desirable discharge intermediates. As anticipated, the battery maintained an attractive cycling life of 237 cycles, even at a superhigh current density of 3000 mA g^−1^.^[^ ^13^ ^]^ Further expanding the design concept, a crystalline nickel sulfide/amorphous nickel phosphide (NiS/NiPO) heterostructure showed high accessibility to guide the formation of thin‐film Li₂O₂.^[^ ^15^ ^]^ By precisely modulating sulfur content, the electronic structure of the heterostructure can be tuned to induce charge redistribution, thereby generating additional active sites that promote the uniform deposition of Li₂O₂. Conventional transition metal‐nitrogen compounds, e.g. M‐N‐C based material, have emerged as a promising catalyst owing to their high ORR activity.^[^ ^19^, ^20^, ^21^ ^]^ For example, a cobalt single‐atom catalyst with a Co‐N₄ coordination structure, anchored on reduced graphene oxide (Co‐SA‐rGO), was shown to catalyze the direct 4e⁻ pathway, achieving an exceptional discharge potential of 2.83 V and a remarkable discharge capacity of 12760.8 mAh g⁻¹.^[^ ^22^ ^]^

Apart from the high efficiency catalyst as discussed above, a rationally designed porous carbon architecture that could facilitate mass transport for oxygen/ ions/electrons and afford sufficient space to accommodate discharge products is also particularly required to achieve high capacity in a Li‐O_2_ battery. Herein, we report the rational design and fabrication of a Co‐N_x_/Co (111) decorated freestanding N‐doped hierarchical carbon framework (Co‐N_x_/Co@NHCF) via a two‐step electrodeposition strategy for application as a cathode in Li‐O₂ batteries. Crucially, density functional theory calculations reveal that the incorporated Co (111) facet can effectively regulate the electronic distribution of N‐carbon, optimize the adsorption of desirable intermediates, and eventually facilitate oxygen reduction reaction/oxygen evolution reaction kinetics. N‐coordinated metal‐atom M‐N4 (M = Co/Ni) catalysts on carbon materials have demonstrated a superior advantage in accelerating the ORR/OER process and inducing uniform Li_2_O_2_ deposition in Li‐O_2_ batteries. Meanwhile, the catalytic impact of Co facilitated the in situ growth of ultrafine carbon nanotubes on the nitrogen‐doped carbon fibers, constructing a 3D hierarchical porous network, which not only ensures superior electronic conductivity but also facilitates efficient oxygen ingress and electrolyte diffusion, while providing ample space for discharge product accommodation. As a result, Co‐N_x_/Co@NHCF catalyzed LOBs delivered a high discharge/charge capacity of 6.15/ 6.22 mAh cm^−2^ with a columbic efficiency of 98.9%, along with a high rate cycling of 700 h at 0.3 mA cm^−2^. This work provides valuable instruction for rational cathode design for high‐performance LOBs via optimization of the crystal structure and the adsorption of intermediates.

## 2 Results and Discussion

### 2.1 Material Synthesis and Characterization

**Figure 1:**
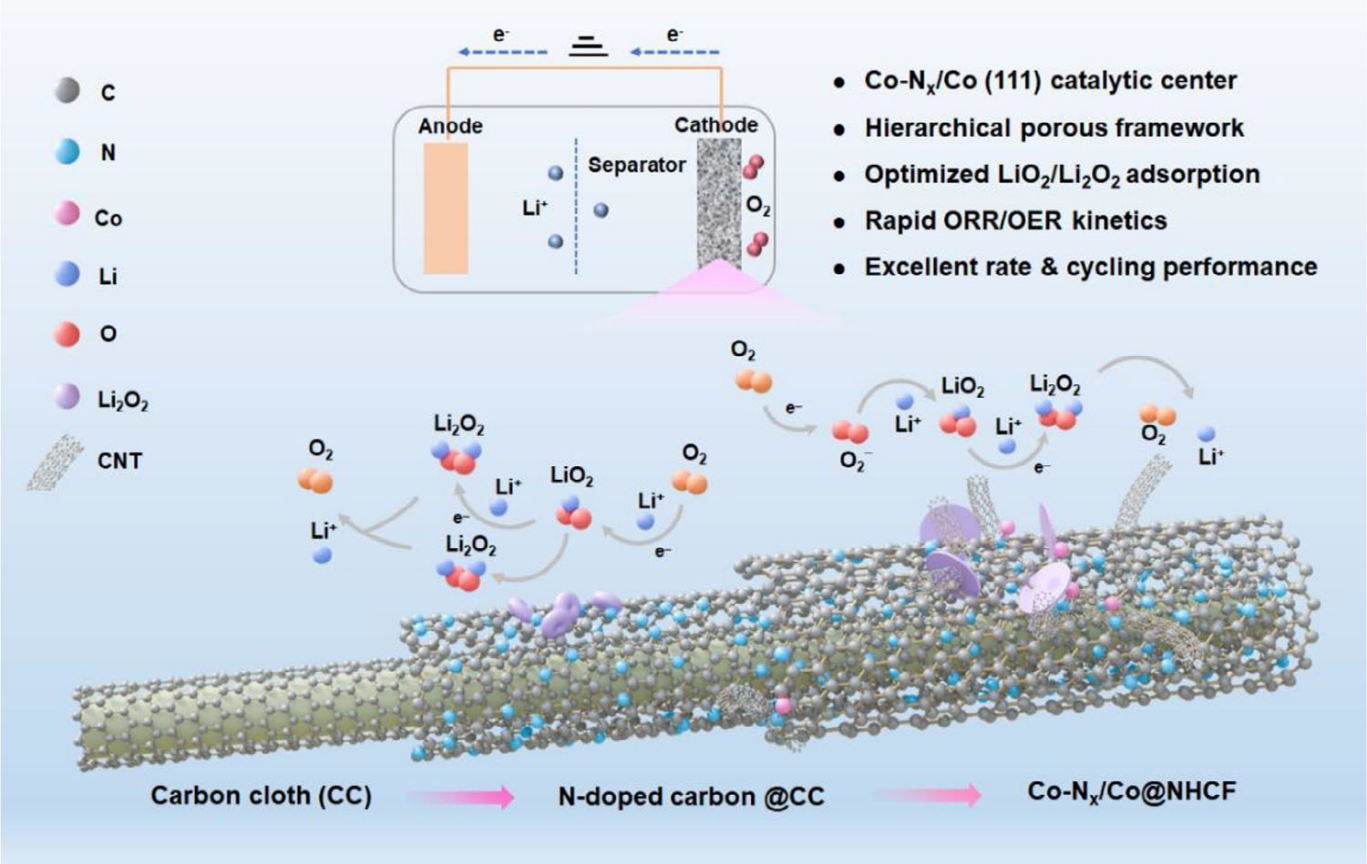
Schematic illustration of the catalytic mechanism toward ORR/OER of Co‐N_x_/Co@NHCF.

A polypyrrole (PPy) fiber network was electrochemically deposited onto carbon cloth (CC) via cyclic voltammetry (CV), yielding PPy@CC. The carbon fibers are uniformly wrapped with PPy nanofibers (Figure S1, Supporting Information). Subsequent high‐temperature calcination under N_2_ atmosphere transformed the PPy network into an N‐doped carbon nanofiber (NCF@CC) framework with high conductivity. The porous fibrous structure was well maintained after the pyrolysis process, as is evidenced by SEM (Figure S2, Supporting Information). Subsequently, cobalt nanoparticles were uniformly deposited onto the NCF@CC substrate via potentiostatic electrodeposition in a 0.1 m Co (NO_3_)_2_ aqueous solution. Further nitrogen doping under thermal treatment facilitated the formation of highly active Co‐N moieties and Co ultrafine clusters, ultimately yielding a freestanding, hierarchically porous structured electrocatalyst (Co‐N_x_/Co@NHCF) with enhanced catalytic properties (**Figure** **1**).

**Figure 2:**
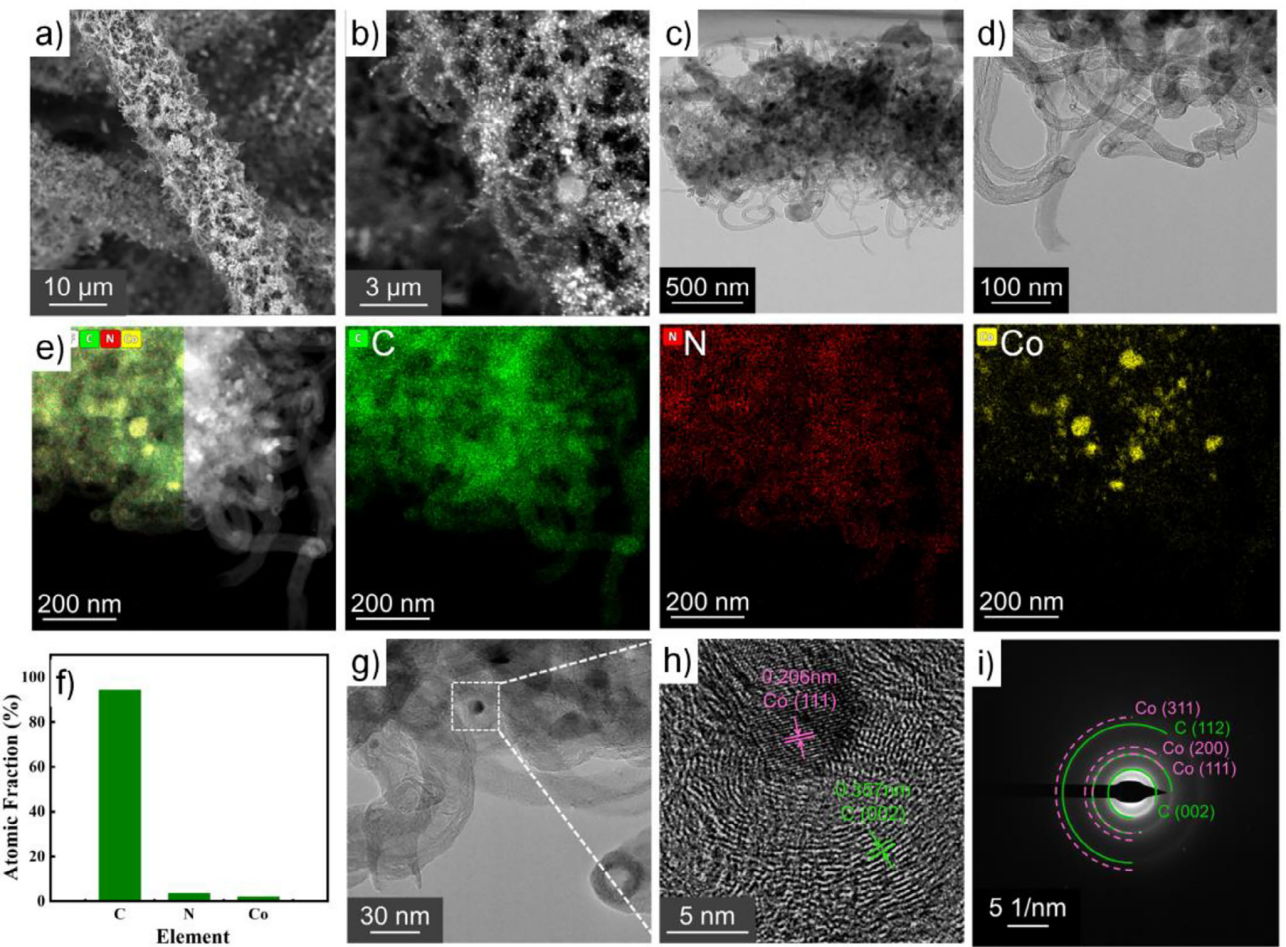
a,b) SEM, c,d) TEM, e) EDS images and corresponding f) atomic fraction diagram, g,h) HR‐TEM images and i) SAED pattern of Co‐N_x_/Co@NHCF.

To elucidate the microstructure and elemental distribution of the Co‐N_x_/Co@NHCF composite, comprehensive physicochemical analyses were conducted. Scanning electron microscopy (SEM) imaging revealed a highly uniform encapsulation of individual carbon fibers by the carbonized polypyrrole (PPy) network, yielding an interconnected porous framework on the carbon substrate (**Figure** **2**a). This hierarchical architecture not only enhances the triple‐phase (solid‐liquid‐gas) reaction interface but also accommodates efficient storage and diffusion of discharge products. Higher‐magnification SEM (Figure 2b) identified ultrathin filamentous nanostructures protruding from the CNF surfaces. Further insights were obtained through transmission electron microscopy (TEM), which unveiled the presence of hollow tubular nanostructures (Figure 2c,d), uniformly dispersed across the CNF matrix and consistent with the morphology of carbon nanotubes (CNTs). This observation aligns with prior reports demonstrating the catalytic role of Co in templating CNT growth during high‐temperature pyrolysis, particularly in the presence of nitrogen‐rich precursors such as melamine.^[^ ^23^, ^24^, ^25^, ^26^, ^27^ ^]^ Elemental mapping via energy‐dispersive X‐ray spectroscopy (EDS‐TEM) confirmed the predominant carbonaceous nature of the nanotubes, corroborating their identification as CNTs (Figure 2e). Notably, C, N, and Co were homogeneously distributed throughout the composite, though localized Co agglomerates were occasionally observed. Due to the more negative formation energy, N preferentially bonded with Co, N doping was performed both before and after Co deposition to obtain both C‐N and Co‐N_x_ species.^[^ ^28^ ^]^ Through repeated EDS mapping, quantitative analysis of the Co element further revealed the coexistence of Co‐N_x_ (0 < x < 4) moieties, while other nitrogen configurations (e.g., pyridinic N and graphitic N) were also found to coexist (Figure 2f; Table S1, Supporting Information), underscoring the complex coordination environment of N within the material. The lattice spacing in the HR‐TEM display reveals lengths of 0.206 and 0.357 nm (Figure 2g,h), representing the (111) crystallographic plane of Co and the (002) crystallographic plane of C, respectively. Such a result agrees well with the selected area electron diffraction (SAED) observation.^[^ ^29^ ^]^

**Figure 3:**
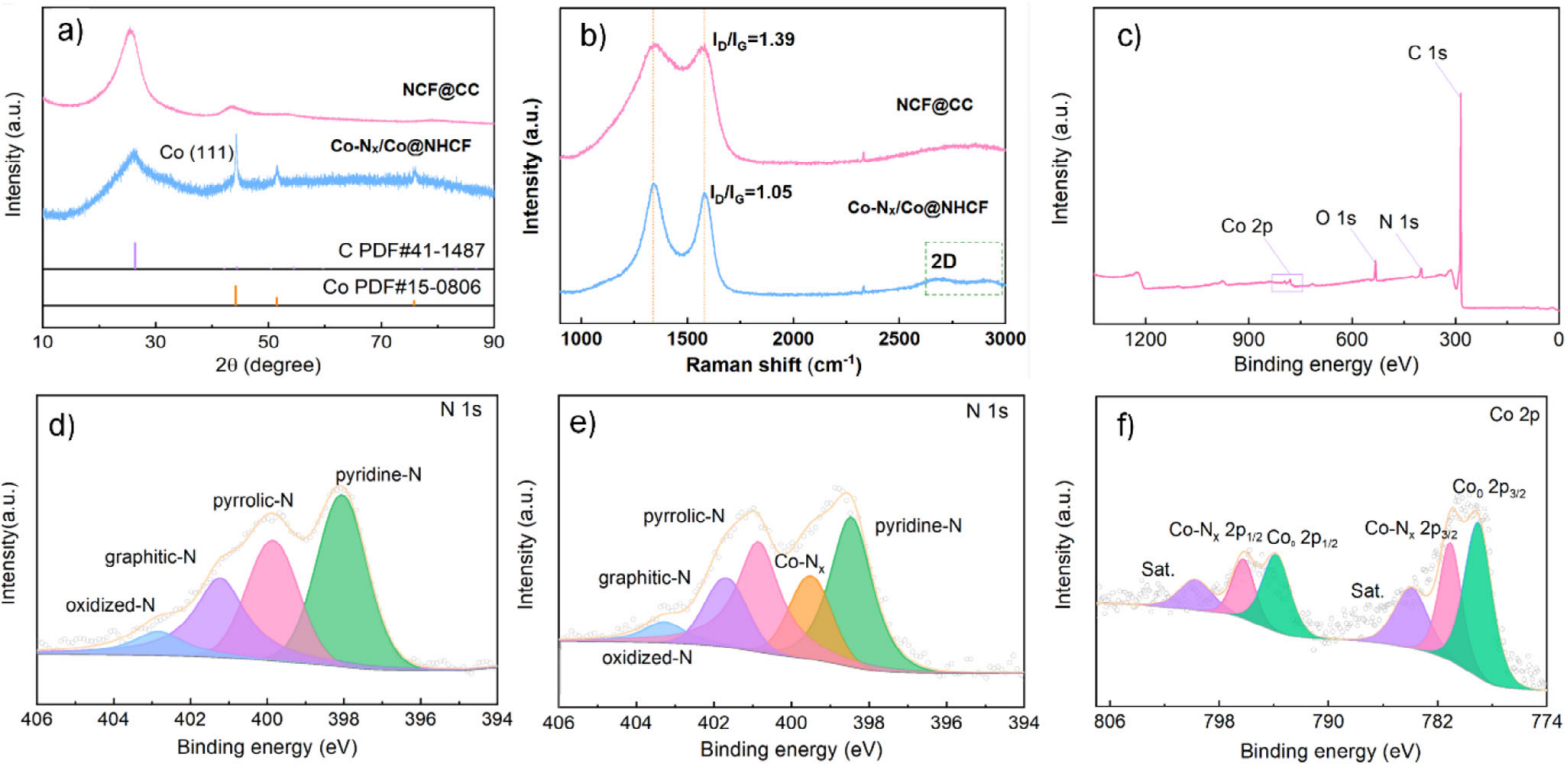
a) XRD patterns, b) FTIR patterns and c) **Raman patterns** of NCF@CC and Co‐N_x_/Co@NHCF, d) XPS spectra for the survey scan and high‐resolution e) N 1s, (f) **Co 2p**.

The crystalline structures of NCF@ CC and Co‐N_x_/Co@NHCF were investigated by X‐ray diffraction (XRD) (**Figure** **3**a). For NCF@ CC, the characteristic diffraction peaks at 26.1° and 43.7° can be indexed to the (002) and (100) crystallographic planes of graphitic carbon (PDF 41–1487), confirming the formation of a well‐defined carbon framework. Upon incorporation of cobalt, the composite exhibits additional Bragg reflections at 44.9°, 52.0°, and 76.6°, which are assigned to the (111), (200), and (220) planes of metallic cobalt (space group Fm‐3m), respectively. These results unambiguously verify the successful in situ formation of crystalline Co nanoparticles within the N‐doped carbon matrix, which is well consistent with the previous reports.^[^ ^24^, ^30^, ^31^, ^32^ ^]^

Raman spectroscopy was employed to investigate the structural information of carbon frameworks in NCF@CC and Co‐N_x_/Co@NHCF (Figure 3c). Both samples exhibit characteristic vibrational modes at 1348 cm^−1^ (D band) and 1586 cm^−1^ (G band), signifying hexagonally bonded carbon atoms within the graphite network and distorted carbon frameworks at defect sites, respectively.^[^ ^17^, ^29^, ^33^, ^34^ ^]^. Notably, the Co‐N_x_/Co@NHCF composite, subjected to secondary high‐temperature annealing during nitrogen doping, demonstrates significant attenuation of these Raman features with a reduced I_D_/I_G_ intensity ratio. This marked decrease indicates substantial graphitic ordering induced by the synergistic effects of i) cobalt‐catalyzed carbon reorganization and ii) thermal annealing at elevated temperatures, which collectively promote the formation of extended conjugated carbon networks with enhanced electrical conductivity.^[^ ^24^ ^]^ Furthermore, the emergence of a well‐defined 2D band at 2750 cm^−1^ in Co‐N_x_/Co@NHCF suggests the presence of numerous defects in the framework, stemming from both N doping and Co‐induced carbon nanotube generation, which is anticipated to further boost the catalytic activity of the material and enlarge the cycling life of the LOBs.^[^ ^31^, ^34^ ^]^

X‐ray photoelectron spectroscopy (XPS) measurement was employed to systematically probe the surface species, bonding configurations, and valence states of the Co‐N_x_/Co@NHCF composite. The survey spectrum confirms the coexistence of Co, O, N, and C elements (Figure 3d), where the oxygen signal predominantly originates from surface oxidation of metallic Co nanoparticles under ambient conditions.^[^ ^29^, ^34^ ^]^ In the high‐resolution N 1s spectra (Figure 3e), distinct anti‐convolution peaks at 398.53, 399.43, 400.5, 401.33, and 403.31 eV indicate the existence of pyridinic nitrogen, Co‐N, pyrrolic nitrogen, graphitic nitrogen, and pyridinic nitrogen oxides, respectively. These multifaceted nitrogen configurations indicate the successful doping of elemental N, C, and Co elements within the carbon matrix and formation of catalytically active Co‐N moieties, suggesting the establishment of an electron‐rich carbon framework with optimized charge transfer characteristics, which is particularly crucial for enhancing oxygen electrocatalysis in battery applications.^[^ ^31^, ^35^ ^]^ The high‐resolution Co 2p XPS spectrum reveals a mixed valence state (Co⁰/Co^2^⁺) (Figure 3f). The presence of Co^0^ indicates cluster formation through thermal‐induced aggregation, consistent with TEM observations and confirming the metallic cobalt phase predominance at high Co loading. Meanwhile, the Co^2+^ species likely either originates from spontaneous surface oxidation of metallic Co cluster (Co⁰) upon air exposure during measurement or from partial oxidation state induced by charge transfer from Co to N in Co‐N moieties. Additionally, the deconvoluted doublet peaks at 782.32 and 797.01 eV are characteristic of Co‐N coordination configurations, serving as efficient catalytically active sites for the ORR/OER reactions.^[^ ^29^, ^36^, ^37^ ^]^

### 2.2 Electrochemical Performance

**Figure 4:**
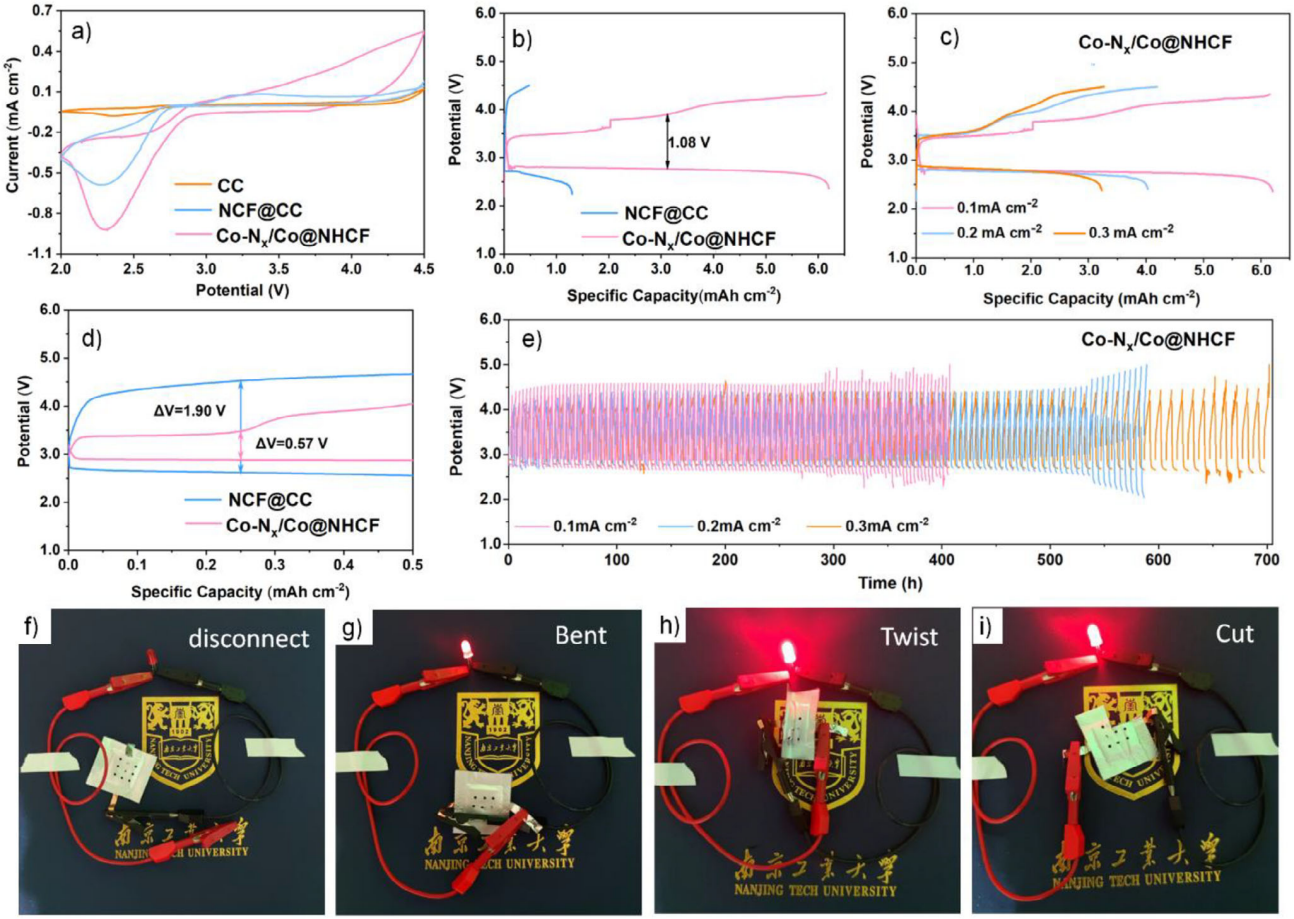
a) CV curves of pristine CC, NCF@CC and Co‐N_x_/Co@NHCF cathode in LOBs, b) initial discharge/charge plots of NCF@CC and Co‐N_x_/Co@NHCF cathode at a current density of 0.1 mA cm^−2^, c) rate capability of Co‐N_x_/Co@NHCF cathode at limited voltage of 2.35–4.35 V, d) the initial discharge/charge curves of the two cathodes with a fixed capacity of 0.5 mAh cm^−2^, e) cycling stability with a fixed capacity of 0.5 mAh cm^−2^ at different currents of the Co‐N_x_/Co@NHCF cathode. Optical images of the flexible LOBs with Co‐N_x_/Co@NHCF cathode powering a commercial red light‐emitting diode at various conditions: f) disconnected, g) bent, h) twist, and i) cut.

Obviously, the carbon cloth matrix shows almost no discernible reduction and oxidation signals from the CV curves (**Figure** **4**a). In comparison, Co‐N_x_/Co@NHCF‐based LOBs represent higher ORR peak current than that of NCF@CC, showing that the construction of the multi‐scale porous network and the introduction of Co could enhance ORR activity. In the subsequent OER process, Co‐N_x_/Co@NHCF shows the highest reaction intensity among the three‐kind of cathodes. Obviously, the presence of Co/Co‐N_x_ dramatically enhances the ORR/OER kinetics and induces a discharge‐recharge voltage gap of only 1.08 V when being initially cycled at a current of 0.1 mA cm^−2^ with the voltage range of 2.35–4.35 V. The NCF@CC sample exhibited a poor discharge/charge capacity of 1.33/0.48 mAh cm^−2^ and a columbic efficiency of 36.09%. In contrast, the Co‐N_x_/Co@NHCF cathode displayed significantly enlarged discharge/charge capacities (6.15/6.22 mAh cm⁻^2^), nearly five times those of NCF@CC, while maintaining a Coulombic efficiency of ≈100% (Figure 4b). The NCF@CC cathode suffered from limited OER catalytic performance, leading to difficulties in decomposing lithium peroxide and the by‐products.^[^ ^38^ ^]^ The Co‐N_x_/Co@NHCF catalyzed battery demonstrates a discharge capacity of 4.07 mAh cm^−2^ with the discharge plateau remaining at 2.75 V when the current density reached 0.2 mA cm^−2^ (Figure 4c). Even at a higher current density of 0.3 mA cm^−2^, the discharge/charge capacities remained impressive at 3.46/ 3.39 mAh cm^−2^, respectively, with the charge capacity exceeding 95% of the discharge capacity at this level. The impressive rate capability and high round‐trip efficiency of the Co‐N_x_/Co@NHCF cathode affirm its potential as a stable and efficient catalytic material for non‐aqueous LOBs.

The particular significance was the outstanding cycling stability demonstrated by the 3D self‐supported Co‐N_x_/Co@NHCF cathode at the cutoff voltage tests. It exhibited the capability to undergo full charge and discharge for over 20 cycles without “Sudden Death”, even at a current density of 0.2 mA cm^−2^ (Figure S3, Supporting Information), demonstrating strong competitiveness from a variety of different dimensions among the currently reported free‐standing catalysts (Figure S4, Supporting Information).^[^ ^39^, ^40^, ^41^, ^42^, ^43^ ^]^ Despite a gradual decrease in discharge/charge capacity and an increase in charge/discharge overpotential with cycling, it remained a high discharge capacity of 2.48 mAh cm^−2^ at the 10th cycle, representing 60.93% of its initial value, showing exceptional cycling stability that was rarely observed in materials of similar types reported to date. To quantitatively evaluate the cycling stability enhancement of the cycling stability of LOBs, galvanostatic charge–discharge tests were performed under 0.1 mA cm⁻^2^ with a capacity limitation of 0.5 mAh cm^−^^2^ (Figure 4e). The charge/discharge potential gap was reduced from 1.90 V of the NCF@CC to 0.57 V of the Co‐N_x_/Co@NHCF cathode, suggesting a robust catalytic effect to facilitate the ORR/OER kinetics of the proposed cathode. The Co‐N_x_/Co@NHCF cathode demonstrated an impressive cycling capability of 700 h at 0.1 mA cm^−2^, affirming its capacity to effectively stabilize the cell cycle life. Moreover, it continued to exhibit stable cycling performance even at higher current densities, achieving 590 and 400 h at 0.2 and 0.3 mA cm^−2^, respectively.

The aforementioned superior electrochemical performance of the Co‐N_x_/Co@NHCF cathode comes from the synergistic effect of the hierarchical porous carbon network and the embedded Co/Co‐N_x_ active catalytic sites. First, the 3D hierarchical fiber network structure facilitated the rapid transfer and diffusion of electrons, ions, and gases during the reaction process, thereby accelerating both the ORR and OER. Second, the homogeneous 3D intertwined hierarchical porous structure provided ample space for the deposition and decomposition of discharge products, which ensured efficient handling of these reactions. More importantly, NC not only offered favorable sites for Co deposition but also collaborated synergistically with Co‐N during the catalytic process. This collaboration induces facial generation and decomposition of Li_2_O_2_, enhancing the ORR and OER catalytic performance. Despite the above advancements, the practical application of Li‐O₂ batteries are still hindered by two fundamental challenges that collectively degrade cyclability. On one hand, the decomposition of the electrolyte induces the accumulation of by‐products, leading to surface passivation and cycle deterioration. On the other hand, the semi‐open cell structure brings the continuous depletion of electrolytes, ultimately resulting in poor circulation and further shortening the cycle life.

A flexible mini pouch Li‐O₂ battery was fabricated employing the as‐synthesized Co‐N_x_/Co@NHCF cathode to evaluate its applicability in wearable and lightweight electronics. Remarkably, the assembled battery successfully powered a red LED under various mechanical conditions, including flat, bent, and even fractured states (Figure 4f–i). These results not only highlight the cathode's exceptional flexibility and operational stability in ambient air but also underscore its robust fault tolerance. The device maintained its functionality despite sustaining significant structural damage, e.g., remaining operational after losing a quarter of its active area during deliberate cutting tests (Figure 6d).

### 2.3 Analysis of the Discharge Product

**Figure 5:**
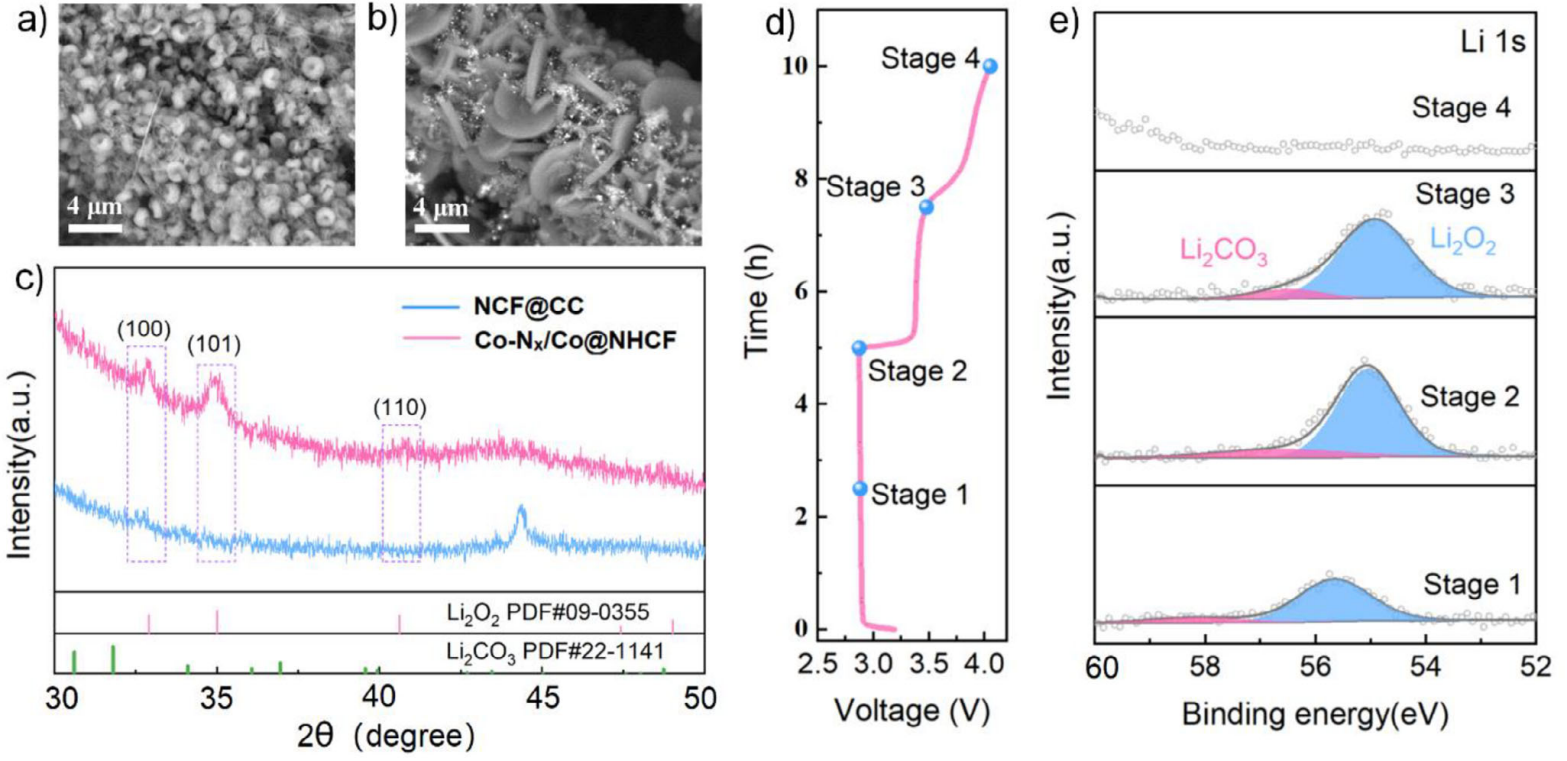
SEM images of the cathode after discharging to 2.35 V of a) NCF@CC and b) Co‐N_x_/Co@NHCF cathode; c) XRD patterns of the discharged two kinds of cathodes; d) Initial discharge/charge plots and e) XPS spectra of high‐resolution Li 1s of Co‐N_x_/Co@NHCF cathode at different discharge/charge states.

In order to clarify the catalytic mechanism of the constructed catalyst, the morphology and crystal structure of the Co‐N_x_/Co@NHCF cathode and its control sample after being fully discharged were investigated by SEM and XRD. The surface of the NCF@ CC electrodes was densely covered by plenty of toroidal‐like discharge products with the diameter of 1–2 µm (**Figure** **5**a), whereas in the case of the Co‐N_x_/Co@NHCF cathode, abundant thin disc‐shaped products with a thickness of only 200 nm were embedded in the porous carbon network rather than merely passivating the surface (Figure 5b). The loose morphology after discharge observed in the Co‐N_x_/Co@NHCF cathode facilitates the transfer of electrons and ions during the subsequent charge process, which is favorable to the decomposition of the discharge product. Additionally, the flaky‐like product is more prone to be decomposed compared to the abacus dense ones, which provides firm evidence of the reduced overpotential upon charging. XRD pattern of the discharged NCF@CC exhibited the typical crystal structure of Li_2_O_2_, with exposed (100), (101), and (110) facets (Figure 5c). However, a rather lower peak intensity of the (100) diffraction was observed in the case of the discharged Co‐N_x_/Co@NHCF, demonstrating poor crystalline of the formed Li_2_O_2_. Previous reports indicated that weakly crystalline or amorphous discharge product usually decomposes more readily at lower overpotentials than their crystalline state.^[^ ^5^, ^11^ ^]^

To deeply analyze the evolution of surface composition on the Co‐N_x_/Co@NHCF cathode during a full cycle, XPS characterization at different discharge/charge depths was employed (Figure 5d,e). The obvious Li 1s peak at 56.5 eV after half‐discharge (stage 1) could be ascribed to the mixed formation of Li‐deficient phase Li_2‐x_O_2_ and Li_2_O_2_. It has been reported that Li_2‐x_O_2_ could be generated during the formation/ decomposition process of Li_2_O_2_.^[^ ^44^ ^]^ With the discharge process deepening to stage 2, pure Li_2_O_2_ was formed as the main product with a Li 1s peak position of 55.2 eV. In the process of recharge, Li_2_O_2_ was gradually decomposed and fully vanished in stage 4, indicating the highly reversible catalytic effect of the as‐prepared cathode. One should note that the weak peak signal of Li_2_CO_3_ during the whole discharge process is induced by the decomposition of the ether‐based electrolyte and the probable reaction between Li_2_O_2_ and the carbon substrate.^[^ ^7^, ^45^, ^46^ ^]^ Nevertheless, although the peak representing Li_2_CO_3_ remains visible in stage 3, it totally disappeared at the end of the recharge process, suggesting that the Co‐N_x_/Co@NHCF cathode could also effectively catalyze the decomposition of Li_2_CO_3_. The above results demonstrate that the designed Co‐N_x_/Co@NHCF could serve as a bifunctional catalyst for LOBs. Its unique hierarchical fiber porous structure, coupled with abundant Co‐N catalytic sites, could effectively regulate the morphology distribution and structure of discharge products, thereby enhancing the LOBs capacity, rate capability, and cycling stability.

**Figure 6:**
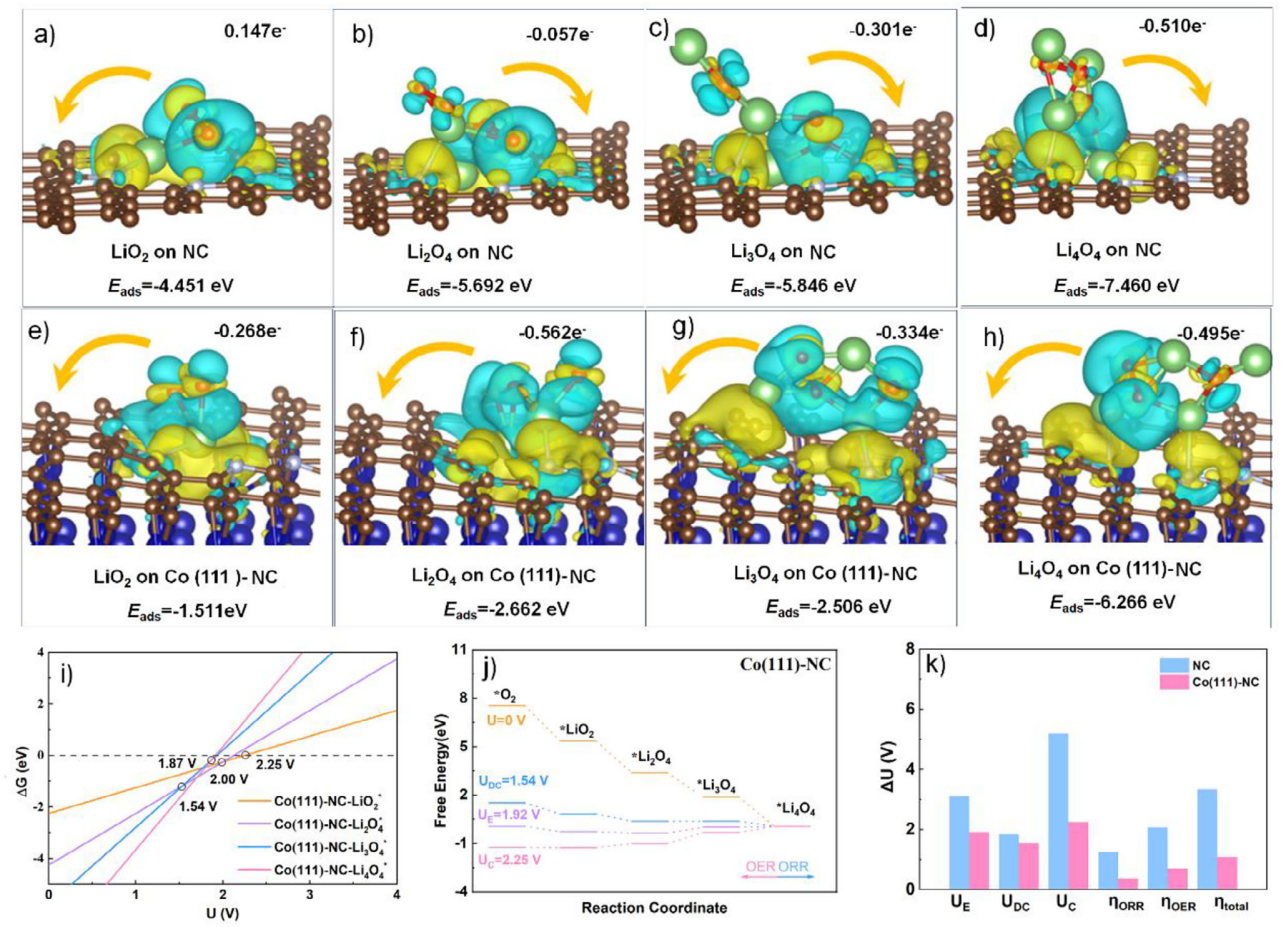
Charge density difference curves of adsorbates (LiO_2_^*^, Li_2_O_4_^*^, Li_3_O_4_^*^, and Li_4_O_4_^*^) on NC (a–d) and Co (111)‐NC (e–h) planes. The yellow and blue areas represent the electron depletion and accumulation, respectively. i) Phase diagram and j) free energy diagram of the ORR/OER reactions on Co (111)‐NC plane, k) overpotentials of ORR/OER processes with Li_4_O_4_ during cycling.

Density functional theory (DFT) calculations were further performed to clarify the influence of the introduction of Co (111) on the binding barrier between the discharge intermediates and catalyst surfaces. Besides, the relative electrocatalytic mechanism of the discharge product could also be revealed. The exposed Co (111) facet and the nitro‐doped carbon (NC) were chosen as the models to contribute planes. The charge density gap and Bader charge transfer of the four kinds of oxygen intermediates adsorbed on the NC matrix were analyzed before and after the Co (111) was embedded, as illustrated in **Figure** **6**a–h. The adsorption energies (Δ*E*_ads_) of the discharged intermediates displayed in Figure 6a–h play critical roles in influencing the electrocatalytic performances of the catalyst in LOBs. Obviously, the Co (111)‐NC exhibits a rather weak binding interaction with LiO_2_^∗^(−1.511 eV), whereas a strong adsorption energy (−4.451 eV) was observed between pure NC and LiO_2_^∗^. As a result, the Li_2_O_2_ morphology could be well regulated by the introduction of the Co (111) facet.^[^ ^12^, ^13^ ^]^ In addition, phase diagrams were calculated to verify the electrochemical stability of the possible discharged intermediates (Figure 6i; Figure S5, Supporting Information). It is illustrated that all LiO_2_, Li_2_O_4_, Li_3_O_4_, and Li_4_O_4_ species can be produced during the ORR process spontaneously.^[^ ^13^, ^14^ ^]^

In a Li‐O_2_ battery, the four reaction step process in below could demonstrate the step‐by‐step discharge process: i) (Li^+^ + e^−^) + O_2_ → LiO_2_^∗^, ii) LiO_2_^∗^  + (Li^+^ + e^−^) +O_2_→ Li_2_O_4_^∗^, iii) Li_2_O_4_^∗^  + (Li^+^ +  e^−^) → Li_3_O_4_^∗^, and iv) Li_3_O_4_^∗^ + (Li^+^ + e‐) → Li_4_O_4_^∗^, where ^*^ represents the adsorbed species on the cathode surfaces.^[^ ^14^ ^]^ The definitions of the overpotential are as follows: η_ORR_  = U_0_ – U_DC_, η_OER_  = U_C_ – U_0_, while η_TOT _ = η_OER_ + η_ORR_, respectively. In detail, U_0_ represents the voltage at which the energy charge of the above four e‐ process is zero. U_DC_ represents the discharge potential and U_C_ refers to the charge potential. The Gibbs free energy at different potentials on the Co (111)‐NC and pure NC during ORR and OER are calculated (Figure 6j; Figure S6, Supporting Information). The charging and discharging overpotentials of the two kinds of catalysts are summarized and compared in Figure 6k. It's obvious that Co (111)‐NC exhibits much lower charge and discharging overpotentials than those of NC, indicating that the introduction of the Co (111) facet can remarkably facilitate the ORR/OER kinetics. Herein, we demonstrated that the introduction of Co (111) facet into the NCF matrix tailors the adsorption capacities of the discharged intermediates and optimizes the ORR/OER kinetics.

## 3 Conclusion

In summary, a Co‐N_x_/Co (111) embedded hierarchical porous carbon framework was developed as a durable cathode for aprotic Li‐O_2_ batteries. The Co‐N_x_/Co@NHCF cathode displayed enlarged discharge/charge capacities of 6.15/ 6.22 mAh cm^−2^ with a columbic efficiency of 98.9%, almost five times that of NCF@CC. Excellent rate performance and remarkable cycling stability were also achieved. Additionally, multiply experimental analysis together with theoretical simulations confirm that the as‐prepared architecture can serve as a bifunctional catalyst toward the rapid formation and decomposition of Li_2_O_2_. This study verifies that the Co‐N_x_/Co active center could significantly affect the intrinsic affinity between the discharged intermediates and the cathode surface, which provides valuable guidance in the rational design of efficient catalysts for LOBs.

## 4 Experimental Section

### Preparation of NCF@CC

Carbon cloth (CC) underwent ultrasonic cleaning with acetone (CH_3_COCH_3_) and deionized water for half an hour each. Subsequently, the CC was immersed in nitric acid within a reactor and hydrothermally treated at 100 °C for one and a half hours for activation. The activated CC was rinsed thoroughly with deionized water until it featured a neutral PH value. Eventually, the CC was dried in an oven at 60 °C overnight.

The PPy@CC precursor was initially prepared using the electrodeposition strategy. The activated CC (2 × 4 cm^2^), a saturated calomel electrode (SCE) clamped with a platinum electrode and carbon rods were employed as the working electrode, reference electrode, and counter electrode, respectively. A solution composed of 0.851 g of lithium perchlorate (LiClO_4_) and 1.696 g of anhydrous sodium carbonate (Na_2_CO_3_) dissolved in 80 mL of deionized water was stirred until complete dissolution. Subsequently, 0.8 mL of pyrrole was added, and the stirring continued for 2 h to obtain a clarified and transparent electrodeposition solution. The three electrodes were assembled into an electrolyzer containing the clarified electrodeposition solution, and the deposition of pyrrole was performed using cyclic voltammetry (CV) and timed‐current (i‐t). The thickness and morphology of the deposited polypyrrole were controlled by manipulating the deposition time of 30 min with an applied potential of 0.85 V and a voltage window of ‐0.8 ‐0.8 V.

The material obtained after deposition was rinsed with deionized water and then dried overnight at 60 °C to produce the PPy@CC precursor. The PPy@CC precursor was then carbonized in N_2_ at 800 °C for 2 h, resulting in the complete carbonization of Ppy nanofibers and yielding the carbon cloth‐loaded N‐doped carbon nanofiber flexible substrate (NCF@CC).

### Preparation of Co‐N_x_/Co@NHCF

The prepared NCF@CC (clamped with a platinum electrode clip) served as the working electrode (green), while the carbon rod functioned as the counter electrode (red), and the saturated mercuric glycol electrode acted as the reference electrode (white). A 0.3 m cobalt (Co(NO_3_)_2_‐6H_2_O) hexahydrate aqueous solution (8.73 g of cobalt hexahydrate dissolved in deionized water, volume fixed to 100 mL) was used as the electrodeposition solution. The three‐electrode deposition device was assembled, and a constant potential deposition was performed by applying a voltage of −1.0 V (vs SCE) on NCF@CC for 15 min. The resulting material was rinsed with deionized H_2_O and then dried at 60 °C overnight, yielding the precursor.

Co‐N_x_/Co@NHCF precursors were placed in a tube furnace, with the side near the inlet rinsed at 60 °C to obtain the Co‐N precursors. Subsequently, the Co‐N_x_/Co@NHCF precursor was placed in a tube furnace, and melamine, weighing ten times the precursor, was added to the side near the air inlet. The entire setup was calcined at 800 °C for 2 h in a nitrogen atmosphere, resulting in the final product, Co‐N_x_/Co@NHCF.

### Physical Characterization

The morphological information of the nanofibrous precursor, NCF, and Co‐N_x_/Co@NHCF was investigated based on a field‐emission scanning electron microscope (FESEM, S‐4800) together with a transmission electron microscope (TEM, JEM‐2100F). X‐ray diffraction (XRD, SmartLab 3KW) with a Cu K target was used to characterize the sample. The compositions and components of the sample were analyzed by X‐ray photoelectron spectroscopy (XPS, Thermo ESCALAB 250XI).

### Electrochemical Characterization

CR2032 coin cells with holes on the cathode side were used as Li‐O_2_ batteries to measure the electrochemical performance. The cells were fabricated in an Ar‐filled glovebox with H_2_O < 0.1 ppm and O_2_ < 0.1 ppm. Lithium foil serves as the anode, free‐standing Co‐N_x_/Co@NHCF (or NCF@CC) works as the cathode, and glass fiber separator (Whatman, GF/F) serves as the separator. 1 m Lithium bistrifluoromethane sulfonimide (LiTFSI) (AR, 99.9%, Macklin) in tetraethylene glycol dimethyl ether (TEGDME, 99.0%, Aladdin) was used as the electrolyte. A multichannel battery testing system (LAND CT2001A) was used to perform galvanostatic discharge/charge tests.

### Theoretical Calculations

Vienna Ab initio Simulation Packages (VASP) with the projector‐augmented wave method were used to perform the DFT theoretical calculations.^[^ ^47^, ^48^ ^]^ The generalized gradient approximation strategy was employed to conduct the calculations.^[^ ^49^, ^50^ ^]^ The cutoff and the convergence criterion for the total energy of the plane wave were set to 450 and 10^−5 ^eV, respectively. The force convergence criterion for all calculations was set to 0.02 eV Å^−1^. The Brillouin zone was sampled with a G‐centered Monkhorst‐Pack k‐point 1×1 mesh.^[^ ^51^ ^]^ A vacuum layer of 15 Å along the *z*‐axis was used for all slab models to avoid periodic structural interactions. The bottom four layers of atoms for the Co (111)‐NC slab models were fixed to simulate the bulk material. The atomic charges and electron‐transfer processes were analyzed by the Bader charge.^[^ ^52^ ^]^

## Conflict of Interest

The authors declare no conflict of interest.

## Supporting information



Supporting Information

## Data Availability

The data that support the findings of this study are available in the supplementary material of this article.
