# Supplementary material for: Fast Li₂O₂ Electrochemistry Enabled by Co‐Nx/Co (111) with Optimized Intermediate Adsorption
Source: Adv Sci (Weinh). 2025 Jul 25;12(39):e10256. doi: 10.1002/advs.202510256 (PMC12533364; doi:10.1002/advs.202510256)
Supplement: Supplementary file 1 — Supporting Information [file ADVS-12-e10256-s001.docx]

**Supporting Information**

**Fast Li₂O₂ Electrochemistry Enabled by** **Co-N_x_/Co (111) with Optimized Intermediate Adsorption**

Lili Liu*, Chen Wang, Luxin Zhao, Yayun Xiao, Weiwei Fang, Lanling Zhao*, Faxing Wang and Yuping Wu*


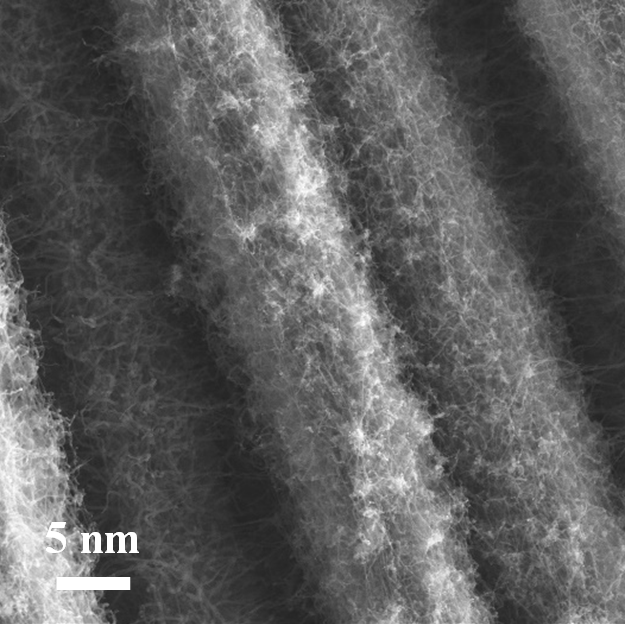


Figure S1. SEM image of CC@Ppy prepared by electrodeposition with the CV program run 30 cycles, and the i-t program run at 0.85V for 30min.


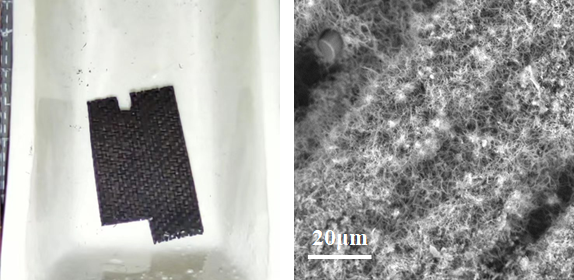


Figure S2. Optical and SEM images the NCF@CC.


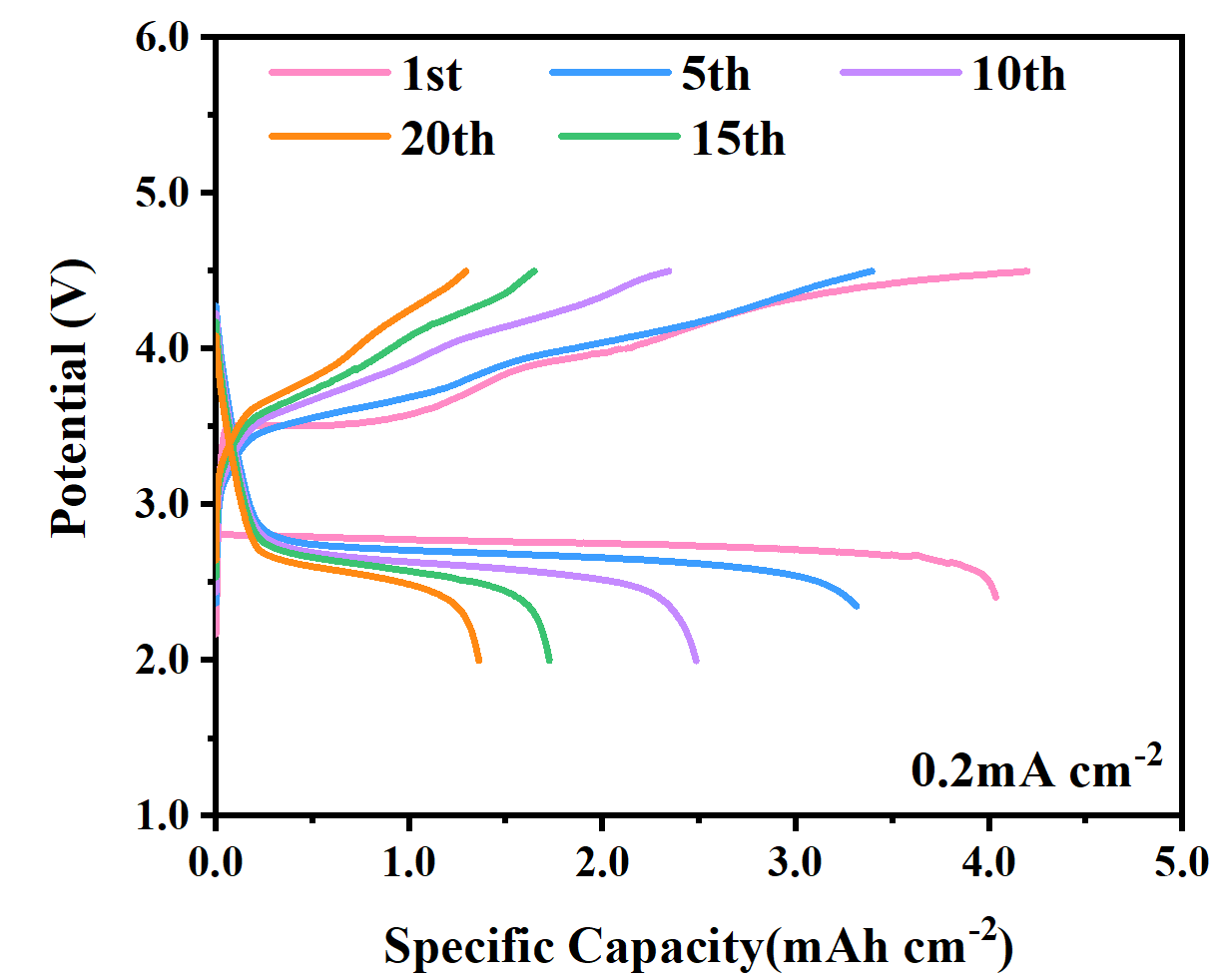


Figure S3. Cycling stability of the Co-N_x_/Co@NHCF cathode at a current density of 0.2 mA cm^-2^.


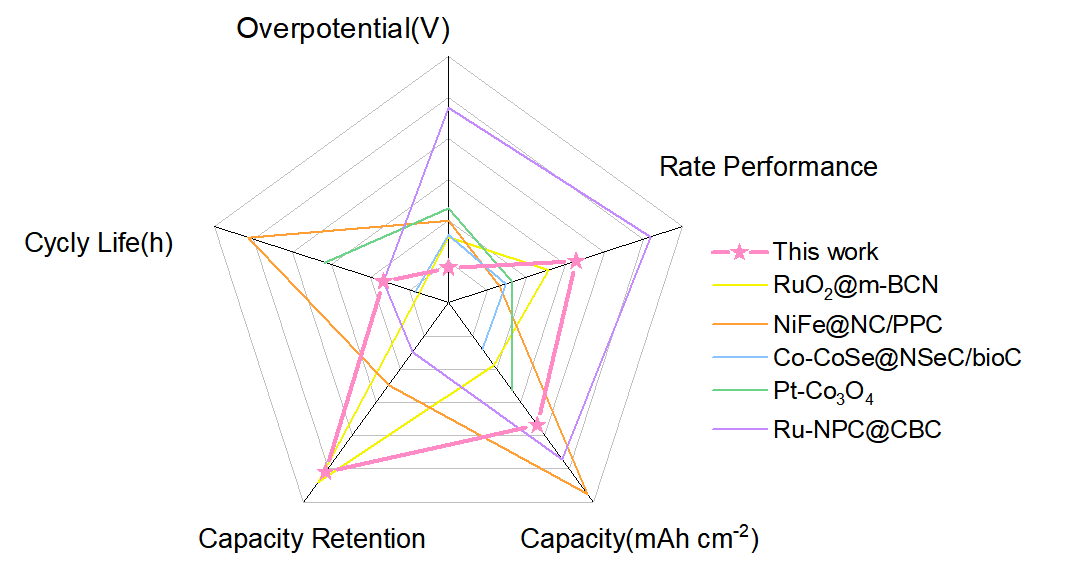
Figure S4. Performance comparison of Co-N_x_/Co@NHCF cathode with some catalysts.


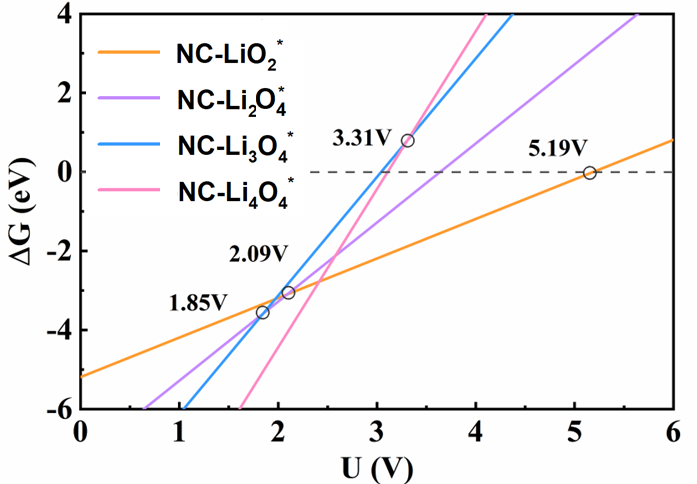


Figure S5. Phase diagram of the cathode reactions on NC plane.


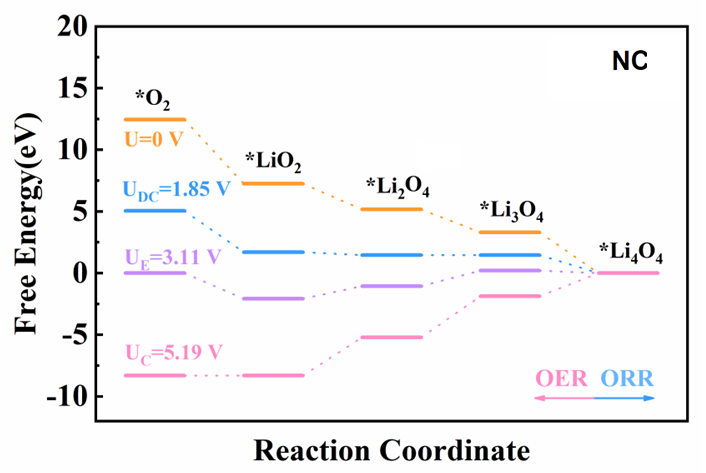


Figure S6. The Gibbs free energy at diﬀerent potentials on the NC plane during ORR and OER.

The adsorption energies of different adsorption intermediates were obtained from the following equation^[1]^:

E*_ads_*=E*_adsorbate/substrate_*-E*_adsorbate_*-E*_substrate_*

E*_adsorbate/substrate_*, E*_adsorbate_* and E*_substrate_* are the total energy of the intermediate and substrate, the intermediate, and the isolated catalyst surface energy.

Besides, the phase diagrams in Figure 6i and Figure S6 (Supporting Information) were obtained. Then reaction pathways can be estimated as follow^[2]^:

ΔG = ΔG^0^-*meU*

*m*, *e*, and *U* are the charge-transfer number, electron charge, and applied voltage, respectively.

The definitions of the overpotential are as follows^[3]^:

η_ORR_ = U_0_-U_DC_

η_OER_ = U_C_-U_0_

η_TOT_ = η_OER_+η_ORR_

**Table S1.** Different mapping data:

| **Element** | **M1 Atomic Fraction (%)** | **M1 Atomic Fraction (%)** |
| --- | --- | --- |
| C | 94.44 | 92.95 |
| N | 3.53 | 4.96 |
| Co | 2.03 | 2.09 |

The adsorption energies of different adsorption intermediates were obtained from the following equation^[1]^:

E*_ads_*=E*_adsorbate/substrate_*-E*_adsorbate_*-E*_substrate_*

E*_adsorbate/substrate_*, E*_adsorbate_* and E*_substrate_* are the total energy of the intermediate and substrate, the intermediate, and the isolated catalyst surface energy.

Besides, the phase diagrams in Figure 6i and Figure S3 (Supporting Information) were obtained. Then reaction pathways can be estimated as follow^[2]^:

ΔG = ΔG^0^-*meU*

*m*, *e*, and *U* are the charge-transfer number, electron charge, and applied voltage, respectively.

The definitions of the overpotential are as follows^[3]^:

η_ORR_ = U_0_-U_DC_

η_OER_ = U_C_-U_0_

η_TOT_ = η_OER_+η_ORR_

**Table S2.** Electrode potentials (U_DC_, U_0_ and U_C_) and overpotentials (η_TOT_, η_OER_ and η_ORR_) for NC and Co (111)-NC:

|  | U_DC_(V) | U_0_(V) | U_C_(V) | η_TOT_(V) | η_OER_(V) | η_ORR_(V) |
| --- | --- | --- | --- | --- | --- | --- |
| NC | 1.85 | 3.11 | 5.19 | 3.34 | 2.08 | 1.206 |
| Co (111)-NC | 1.54 | 1.92 | 2.25 | 1.09 | 0.71 | 0.38 |

[1] HAN X, ZHAO L, LIANG Y, et al. Interfacial Electron Redistribution on Lattice‐Matching NiS_2_/NiSe_2_ Homologous Heterocages with Dual‐Phase Synergy to Tune the Formation Routes of Li_2_O_2_ [J]. Advanced Energy Materials, 2022.

[2] HAN X, ZHAO L, WANG J, et al. Delocalized Electronic Engineering of Ni5P4 Nanoroses for Durable Li–O_2_ Batteries [J]. Advanced Materials, 2023, 35(35).

[3] ZHOU Z, ZHAO L, WANG J, et al. Optimizing E_g_ Orbital Occupancy of Transition Metal Sulfides by Building Internal Electric Fields to Adjust the Adsorption of Oxygenated Intermediates for Li‐O_2_ Batteries [J]. Small, 2023, 19(41).
